# On Magnetic Models in Wavefunction Ensembles

**DOI:** 10.3390/e25040564

**Published:** 2023-03-25

**Authors:** Leonardo De Carlo, William D. Wick

**Affiliations:** 1 Scuola Normale Superiore, Piazza dei Cavalieri, 7, 56126 Pisa, Italy; 2 Department of Economics and Finance, Luiss Guido Carli, Viale Romania, 32, 00197 Rome, Italy; 3 Independent Researcher, Seattle, WA 98119, USA

**Keywords:** quantum magnetism, wavefunction ensembles, large deviations

## Abstract

In a wavefunction-only philosophy, thermodynamics must be recast in terms of an ensemble of wavefunctions. In this perspective we study how to construct Gibbs ensembles for magnetic quantum spin models. We show that with free boundary conditions and distinguishable “spins” there are no finite-temperature phase transitions because of high dimensionality of the phase space. Then we focus on the simplest case, namely the mean-field (Curie–Weiss) model, in order to discover whether phase transitions are even possible in this model class. This strategy at least diminishes the dimensionality of the problem. We found that, even assuming exchange symmetry in the wavefunctions, no finite-temperature phase transitions appear when the Hamiltonian is given by the usual energy expression of quantum mechanics (in this case the analytical argument is not totally satisfactory and we relied partly on a computer analysis). However, a variant model with additional “*wavefunction energy*” does have a phase transition to a magnetized state. (With respect to dynamics, which we do not consider here, wavefunction energy induces a non-linearity which nevertheless preserves norm and energy. This non-linearity becomes significant only at the macroscopic level.) The three results together suggest that magnetization in large wavefunction spin chains appears if and only if we consider indistinguishable particles and block macroscopic dispersion (i.e., macroscopic superpositions) by energy conservation. Our principle technique involves transforming the problem to one in probability theory, then applying results from large deviations, particularly the Gärtner–Ellis Theorem. Finally, we discuss Gibbs vs. Boltzmann/Einstein entropy in the choice of the quantum thermodynamic ensemble, as well as open problems.

## 1. Motivations, Results and Organization of the Work

In recent decades not only has entanglement been observed both at the microscopic level [1] and in “semi-macroscopic” objects [2,3,4] (even at room temperature [5]), but remarkably also superposition states became fundamental to describe not only atomic-level observations [6,7,8] but also large atom complexes [9,10,11,12], for which they observed “cat” states, i.e., states where the center of mass (COM) displayed spatial dispersion or interference patterns. These developments are leading to an experimental program to locate the classical-quantum boundary and the underlying mechanism [13].

In our view, the above-mentioned phenomena can be understood only by considering the wavefunction as a configuration of matter, encoding a physical state in a high-dimensional Hilbert space whose effect is felt in the ordinary three-dimensional space, as Schrödinger originally intended [14]. But this view also implies paradoxes, as pointed out by Schrödinger himself [15]: the unrestricted linearity of quantum mechanics produces situations where macroscopic objects exhibit COM dispersion. (Maybe this was the biggest handicap of his theory and view that particles are not individuals.) On the other side the unrestricted linearity of quantum mechanics is essential for many of the successful predictions at the atomic–nuclear level, but today there is a shared opinion [13] that quantum mechanics could have a size limit to its application. This idea and the mentioned observations about superposition states raise the possibility of deviations from Schrödinger’s equation. This research line was encouraged by S. Weinberg in a recent popular account [16] where he exposed the struggle of quantum mechanics with the macroscopic world known as the “measurement problem”. He explained that modifications have to be undetectable at atomic–nuclear level and at the same time “eliminate” macroscopic superpositions without granting the apparatus any special status. We mention that Weinberg in 1989 developed a mathematical framework [17] to test non-linear generalizations of Schrödinger’s equations at the atomic–nuclear level. Subsequent experiments appeared to rule out the models he examined [18]. (However, models with wavefunction energy escape this net [19].)

Here we consider wave-mechanical configurations for simple spin models with the goal of constructing Gibbs statistical ensembles of magnets containing all the states of the Hilbert space of quantum mechanics. We found it relevant to consider a modification (small for few degrees of freedom and very large for many degrees of freedom in a sense to clarify later) of quantum mechanics represented by an energy giving a “cost” to superpositions, that we call “wavefunction energy” (WFE), thus eliminating the macroscopic dispersion that afflicted the original wave mechanics of Schrödinger. The corresponding dynamical framework is the Hamiltonian one of Weinberg [17] but with a very important difference, observed in [19], that implies conservation of the norm during the evolution. This general set-up and the principles of this proposal will only be briefly described in Section 3.1 since dynamical problems are not in the purview of the present paper.

## 2. Materials and Mathematical Results

In standard quantum mechanics, one first diagonalizes the Hamiltonian to discover the eigenvalues; call them $\left\{E_{n}\right\}$; then, if the energy is ever measured, the result would be to “find” one of the $E_{n}$, and the system jumps into the corresponding eigenstate. (The realist assumption that a system actually exists in one of the eigenstates leads to contradictions whenever certain pairs of observables are involved, such as position and momentum, or energy and time. Hence the reliance on textual formulations involving “finding” rather than “being”. (John Bell made these points eloquently, especially in his last paper “Against ‘measurement”’ [20].)) Thus a thermal ensemble is constructed using these energy-measurement “outcomes” of form:

(1) Fβ..=..Z−1∑nexp{−βEn/kT}<ψn|F|ψn>,

where β is the inverse temperature and $\psi_{n}$ is the eigenstate corresponding to eigenvalue $E_{n}$ in the Hilbert space. In the special case of the Heisenberg model, with Hamiltonian operator expressed in terms of Pauli matrices, where only the z-axis interacts $H=-\;2\;\sum_{i,j;n.n.}^{N}\;\sigma_{z;i}\;\sigma_{z;j}$, the model reverts to the Ising model: interpreting the classical spins $S_{i}$ as pointing up or down the z-axis and as labels of Hilbert space vectors, e.g., in Dirac notation, $|S_{1},\ldots ,S_{N}>$, they already define an eigenbasis of *H*.

In our first attempt to treat wave-mechanical spin models, we restrict to *N* spins $S_{1},\ldots ,S_{N}$ taking values $\pm \frac{1}{2}$ and assume interaction only along the z-axis. The state is not now a spin configuration but a wavefunction on such configurations, i.e., of form $\psi (S_{1},\ldots ,S_{N})$ where each value is a complex number. Thus ψ lies in a space of $2.2^{N}$ real dimensions (with the notation $2.2^{N}$ we mean $2^{N}$ dimensions because of the possible classical configurations and with $2.2^{N}$ we double the dimensions because of the real part and the complex one of a state ψ). The energy of a wavefunction ψ is what is called in standard quantum mechanics “the expected energy”. In particular, the energy E(ψ) contains contributions from all spin configurations (“superpositions”). The Gibbs canonical ensemble is not based on a product measure.

The probability generating function we wish to evaluate now takes the form:

(2) ZN(β;λ1,…,λN)..=..∫||ψ||=1dψexp−βE(ψ)+ΛN(ψ),

where ${\left||\psi |\right|}^{2}=\sum_{S}\;{\left|\psi \left(S_{1},\ldots ,S_{N}\right)\right|}^{2}$ with *S* a classical configuration of spins. The integral is over the unit sphere in the above-stated number of dimensions. The energy is

(3) $$E_{N}\left(\psi \right)=-<\psi |\;E\left(S\right)\;|\psi >=-\sum_{S}\;{\left|\psi \left(S_{1},\ldots ,S_{N}\right)\right|}^{2}\;E\left(S\right),$$

with E(S) a classical spin magnetic energy of order *N* in the configuration *S*, as Curie–Weiss $\frac{1}{N}\;{\left(\;\sum_{i=1}^{N}\;S_{i}\;\right)}^{2}$ or Ising $\sum_{n.n.}S_{i}S_{j}$ (n.n. means nearest neighbor sites *i* and *j*), and

(4) $$\Lambda_{N}\left(\psi ;\lambda_{1},\ldots ,\Lambda_{N}\right)=<\psi |\sum_{i=1}^{N}\;\lambda_{i}\;S_{i}\;|\psi >=\sum_{S}\;{\left|\psi \left(S_{1},\ldots ,S_{N}\right)\right|}^{2}\sum_{i=1}^{N}\;\lambda_{i}\;S_{i}.$$

The sum $\sum_{S}$ in (3) and (4) is the sum over all spin configurations.

The motivation for the base integral over normalized wavefunctions differs from the Copenhagenist, in which ψ represents a probability distribution on spin configurations. Rather, Schrödingerist reasoning is that we do not want to compare states on the basis of normalizations, e.g., one with norm 0.1 and another with norm 100, but solely by their respective energies. Moreover, both linear quantum mechanics and suitable nonlinear generalization [19], preserve the norm, and the latter raises the possibility that the flow is ergodic or chaotic, with a possible interest in a classical justification for the Second Law of Thermodynamics. Thus we would not wish to drop the normalization.

The objective of this work is to understand whether it is possible to construct physically meaningful wave-mechanical magnetic models and to explore the implications. In Section 3 we consider distinguishable particles, namely we take the full $2.2^{N}$-sphere as state space without imposing any exchange symmetry on the wavefunctions. In this case the models will not magnetize (except at 0 temperature): $\lim_{N\to \infty }{\;[\;\{\;|m\left(\psi \right)|>\epsilon \;\}\;]}_{\beta }=0$ for any ϵ, where $m\left(\psi \right)\;=\;<\psi |\;\sum \;S_{i}{/N\;|\psi >\;=\;\sum \;|\psi \left(S\right)|}^{2}\left(\;\sum \;S_{i}\;\right)/N$ and ${\left[\cdot \right]}_{\beta }$ is the ensemble thermal average.

In Section 3.1 we introduce a generalized Hamiltonian framework, as developed in [17,19], assuming a non-linearity that penalizes large superpositions in large objects. This non-linearity makes energetically impossible configurations corresponding to macroscopic cat states. (For a modern mathematical definition of cat states see [19].) Even in this case, with distinguishable particles no phase transition appears (except at zero temperature).

In Section 4 we specialize to the mean field case with exchange symmetry. With this choice, the dimensionality of the Hilbert state space is greatly reduced and the model manifests magnetization at finite temperatures, when wavefunction energy is included. (In this case but without WFE, showing that there is no phase transition assumes some properties of a certain two-argument function for which we provide computer-generated evidence). To our knowledge, this is the simplest wavefunction model in which a phase transition appears.

In Section 5, we discuss the choice of ensembles with uniform measure, as opposed to other possibilities. In particular we introduce a base measure that includes a quantum Boltzmann–Einstein entropy and discuss how the usual ensembles should be recovered in a suitable limit.

The paper is organized as follows: in each section the primary results are described but the mathematical derivations are relegated to Appendix A. Our principle technique involves transforming the problem to one in probability theory, then applying results from large deviations, particularly the Gärtner–Ellis Theorem.

**Definition of phase transition.** In classical spin magnetic models one is interested in whether in the thermodynamic limit (N→∞) there is a spontaneous magnetization at sufficiently low temperature. This means the following. Define the magnetic field generated by the N spins $S_{i}$ as:

(5) M..=..∑i=1NSi.

From the up–down symmetry of E(S) one deduced that ${\left[\;M\;\right]}_{\beta }=0$, so the average is not the relevant question. There are two ways to continue. One can introduce + boundary conditions, say by fixing $S_{1}=S_{N}=+\frac{1}{2}$, breaking the symmetry, and inquire as to whether, below a critical temperature,

(6) $$\lim_{N\to \infty }\;{\left[\;M\;\right]}_{\beta }>0.$$

Another approach is to maintain the “free” boundary conditions and inquire into the behavior of the field per spin, M/N; does it exhibit Central Limit Theorem (CLT) behavior; that is, ≈ O$\left(1/\sqrt{N}\right)$? This implies ${\left[\;|M|\;\right]}_{\beta }\approx \sqrt{N}$. If one interprets “O(N)” as macroscopic, one concludes that spontaneous magnetization did not appear. The CLT would hold if the correlations decayed exponentially:

(7) $${\left[\;S_{i}\;S_{j}\;\right]}_{\beta }\approx \exp\left\{\;-\xi \;d\left(i,j\right)\;\right\};$$

(for some constant ξ>0, where d(i,j) denotes distance between the lattice points labeled *i* and *j*) but not if correlations decayed more slowly or even were bounded away from zero. In that case, assuming below a critical temperature the dispersion

(8) $${\left[\;{\left(\;M/N\;\right)}^{2}\;\right]}_{\beta }\approx \mathrm{constant}>0,$$

one would conclude that the limiting ensemble was a mixture of magnetized states and a phase transition had occurred. Observing the behavior (8) is the concept of phase transition we use throughout the paper, where we replace M/N and ${\left(M/N\right)}^{2}$, respectively, with m(ψ) and $m^{2}\left(\psi \right)$.

## 3. Models with Distinguishable Particles

The starting point, see Appendix A.3, is to replace the random point on the sphere in (2) with

(9) $$\psi \left(S\right)⟶\frac{\chi (S)}{\sqrt{{\sum \;|\chi \left(S\right)|}^{2}}},$$

where $\left\{\;\chi \left(S\right):S=\left(S_{1},\ldots ,S_{N}\right)\;\right\}$ are $2^{N}$ complex, or 2.$2^{N}$ real, numbers distributed as i.i.d. standard (mean zero, norm one) Gaussians. This procedure defines a measure on the sphere and the rotational symmetry of the standard Gaussian measure (covariance matrix: the identity) then assures that we are considering the same distribution of wavefunctions. By this transformation and various lemmas, we will reduce the problems of next sections to finding probabilities of rare events (called “large deviations” theory). Let

(10) m(ψ)=<ψ|∑Si/N|ψ>=∑|ψ(S)|2(∑Si/N);[F(ψ)]β..=..∫||ψ||=1dψexp{−βEN(ψ)}F(ψ)/ZN.

**Theorem 1.**  *Given a magnetic spin energy E(S) of order N, e.g., Curie–Weiss or Ising, in any dimension for any finite temperature (except T = 0) there is no magnetization in the thermodynamic limit:*

(11) $$\lim_{N\to \infty }{\;[\;\{\;|m\left(\psi \right)|>\epsilon \;\}\;]}_{\beta }=0,$$

*for any ϵ>0.*

For the proof, see Math Appendix A.3. This case does exhibit zero-temperature magnetization. Consider Curie–Weiss or Ising and β→∞, the probability distribution becomes concentrated on states of minimal energy. These have the form:

(12) ψ(θ,α)=cos(θ)ψ+..+..sin(θ)e−1αψ−,

where $\psi_{\pm }$ denotes a wavefunction concentrated on all up, respectively all down, spins. Hence at T=0 the magnetization takes the form:

∫0π/2dθf(θ)<ψ(θ,α)|MN|ψ(θ,α)>2..=..14∫0π/2dθf(θ)cos2(2θ)>0.

### 3.1. Models with Wavefunction Energy

We consider a modification of the $E_{N}\left(\psi \right)$ adding nonquadratic terms into the wavefunction. We will refer to these terms as representing “wavefunction energy” (WFE). Our principles to modify the usual quantum Hamiltonian are:

(a) The modification has to be negligible at the microscopic level but becomes large enough at the macroscopic level to block dispersion (macroscopic dispersion of the spins is better explained later);
(b) The norm of ψ and the energy of a closed system have to be conserved (the same for momentum);
(c) No extra terms are added to the evolution of the center of mass $X\left(\psi \right)=<\psi |\frac{1}{N}\sum_{N}^{j=1}x_{j}|\psi >$ of a closed system.

These characteristic are satisfied when the evolution of the quantum state is

(13) $$i\hbar \frac{\partial \psi }{\partial t}=\frac{\partial }{\partial \psi^{*}}E\left(\psi \right).$$

with

(14) E(ψ)..=..EQM(ψ)..+..EWFE(ψ),

(15) $$E_{WFE}\left(\psi \right)=wN^{2}D\left(\psi \right),\;\;D\left(\psi \right):=<\psi \left|\;{\left(\;\overset{N}{\sum_{i=1}}\;O_{i}-<\psi \left|\;\overset{N}{\sum_{i=1}}\;O_{i}\;\right|\psi >\;\right)}^{2}\;\right|\psi >$$

where *w* is a very small constant and the $O_{i}$’s are self-adjoint operators diagonal in the same base as $H_{QM}$, see the proofs in [19]. When in (14) we take w=0 the energy E(ψ) becomes $\langle \psi |H_{QM}|\psi \rangle$ where $H_{QM}$ is the usual quantum Hamiltonian given by the sum of the kinetic and potential terms; taking the derivative with respect to $\psi^{*}$ in (13) one finds the Schrödinger’s equation.

In [19,21] one author explored nonlinear Schrödingerist quantum mechanics as a potential solution to the Measurement Problem: the addition of nonquadratic terms to the Hamiltonian, i.e., the WFE, were proposed to block spatial dispersion in macroscopic objects (and otherwise be too small to matter). The WFE in the present work will be a functional of the wavefunction addressing dispersion of the total spin, instead of center of mass or total momentum (as in [22]). This means $O_{i}=S_{i}$. This choice just reflects the lack of spatial coordinates in our models of magnetism. The straightforward generalization of (16) to models with spatial coordinates is $O_{i}=L_{i}+S_{i}$ in (15) and [*w*] = kg${}^{-1}$m${}^{-2}$. The original proposal [23] is on the momentum $P_{i}$. The general idea behind (15) derived in part from an interview with Hans Dehmelt, see chapter 15 in [24]. He explained to one of the authors that classical physics applies when for some reason wavefunctions cannot spread out (forming cats). For instance, classical-like orbits in electromagnetic traps are due to the trapping fields. The mechanism (15) proposes a universal self-trapping forbidding macroscopic dispersion, so to speak. (The ideal experimental test to falsify WFE is described in [22]).

For our statistical ensembles the first term $E_{QM}\left(\psi \right)$ now incorporates the usual spin–spin and spin–external field interactions, while the second $E_{WFE}\left(\psi \right)$ takes the form:

(16) EWFE(ψ)..=..wN2D(ψ);D(ψ)..=..1N2<ψ|∑Si−<ψ|∑Si|ψ>2|ψ>..=..1N2<ψ|∑Si2|ψ>−<ψ|∑Si|ψ>2.

Here *w* is a positive constant small enough to make the mechanism undetectable at atomic level and we have written the sum of spins rather than *M* to emphasize the interpretation as the “dispersion of the center-of-spin”. We observe that D(ψ) is of order 1 on *cat states*, i.e., close to (the concept can be made precise introducing a suitable spherical distance)

(17) $$\psi =\frac{1}{\sqrt{2}}\psi_{+}+\frac{e^{\sqrt{-1}\alpha }}{\sqrt{2}}\psi_{-},$$

and therefore $E_{WFE}\left(\psi \right)$ of order $wN^{2}$, of order 1/N on *product states*, i.e., close to

(18) P.S...=..ψ:…ψ(S1,…,SN)=∏i=1Nψi(Si),|ψi(1/2)|2+|ψi(−1/2)|2..=..1,…∀i.

and therefore $E_{WFE}\left(\psi \right)$ of order wN, of order $1/N^{2}$ close to *eigenstates* (classical configurations with no superpositions) and therefore $E_{WFE}\left(\psi \right)$ of order *w*. We observe that for the macroscopic magnetization M(ψ)=Nm(ψ) the situation will be opposite, that is it will be very small close to product and cat states while of order *N* close to eigenstates.

Let us first examine T=0. Again, the case reduces to support on wavefunctions of form:

(19) ψ(θ,α)=cos(θ)ψ+..+..sin(θ)e−1αψ−,

plugging into the definition of *D* yields:

(20) $$E_{WFE}\left(\psi \right)=\frac{N^{2}}{4}\;\sin^{2}\left(2\;\theta \right).$$

Since by adding a trivial constant (which does not alter the measure) the usual energy, respectively, for Ising and Curie–Weiss, can be rewritten:

(21) $$E_{QM}\left(S_{1},\ldots ,S_{N}\right)=\sum_{i,j,n.n.}\;{\left(\;S_{i}-S_{j}\;\right)}^{2}\;\mathrm{and}\;N\left(1-m^{2}\left(S\right)\right),$$

both forms of energy have the same sign (positive). So in the presence of the WFE terms the support is reduced to the cases with θ=0 or θ=π/2; hence, the ensemble becomes a mixture of two magnetized states, as in (13). “Classical” behavior is restored.

Thus, the interesting question arises as to whether this conclusion will persist at nonzero temperatures. We observe that the “correlations” suppressed by including $E_{WFE}\left(\psi \right)$ in the Gibbs factor concern single wavefunctions and whether they imply a cat, while the correlations important for magnetization are thermal. For a discussion about why these models can not be regarded as “continuous spin” models, see [25].

In Math Appendix A.3 we show that adding the WFE to distinguishable particles does not change the conclusion about magnetization (namely, none at any finite temperature).

**Proposition 1.**  *For distinguishable particles with Hamiltonian* (14)*, given a magnetic spin energy E(S) of order N, e.g., Curie–Weiss or Ising, in any dimension for any finite temperature (except T = 0) there is no magnetization in the thermodynamic limit:*

(22) $$\lim_{N\to \infty }{\;[\;\{\;|m\left(\psi \right)|>\epsilon \;\}\;]}_{\beta }=0,$$

*for any ϵ>0.*

Namely, even under non-linear modifications, it is not possible to produce phase transitions, which we attribute to entropy swamping energy because of high dimensionality of the models.

## 4. Models Assuming Exchange Symmetry: The Schrödingerist Curie–Weiss Model

As starting point to remove the notion of distinguishable particles we consider the Curie–Weiss energy:

(23) ECW(S)..=..−1N∑i=1NSi2,

where we are going to replace spin configurations by wavefunctions with exchange symmetry; for such a system one should consider “integer spin”, say {−1,0,+1}; see [26,27] for examples of integer spin models. Here we adopt two to learn how to construct some mathematical tools for these wave-mechanical models, reasoning that the lowest possible dimensionality and mean field interaction define the first case to study whether phase transitions appear or not. Models with higher dimensions, meaning higher entropy, or local interactions presumably are even less likely to exhibit such transitions. (Both the concept and the mathematical definition of nearest neighbors for indistinguishable particles appears somewhat problematic in wavefunction models.) In [28], qu-bits are considered with the exchange symmetry. IBM [29] (see references there) says that titanium atoms can represent qu-bits and they project to make a complex of many of them to observe how the collective behavior changes; this might be a situation described by the SCW model.

We define a wave-mechanical model with Hamiltonian (23) and exchange spin symmetry. Note that with two levels the CW energy depends only on the number, call it “*n*”, of “down” spins. Thus we are led to introducing wavefunctions that depend only on “*n*”. On N-dimensional Euclidean space, define, given some $a_{i}>0$:

(24) ∫{∑xi2ai=1}∏i=1NdxiF(x1,…,xN)..=..limσ→0Aellipsoid−1∫∏dxiexp{−(∑xi2ai−1)2/σ2}F(x1,…,xN)/W(σ),

where “$A_{\mathrm{ellipsoid}}$” stands for the surface area of the ellipse $\left\{\;\sum \;x_{i}^{2}\;a_{i}=1\;\right\}$ and

(25) W(σ)..=..∫∏dxiexp{−(∑xi2ai−1)2/σ2}.

Thus we utilize an approximate Dirac delta-function becoming concentrated in the limit on the ellipsoid. Now let us make a change of variables by defining: $y_{i}=x_{i}/\sqrt{a_{i}}$ in (24). A Jacobian derivative of $\prod \sqrt{a_{i}}$ appears in numerator and denominator and so cancels, yielding:

**Lemma 1.**  *Let $A_{\mathrm{sphere}}$ be the “surface area” of the N-sphere. Then:*

∫{∑xi2ai=1}∏i=1NdxiF(x1,…,xN)..=..AsphereAellipsoid∫{∑yi2=1}∏i=1NdyiF(a1yi,…,aNyN).

We want to define an ensemble and corresponding integrals for symmetric wavefunctions. Each such wavefunction is uniquely given by its common value, call it $\hat{\psi }\left(n\right)$, on the set of configurations with *n* “down” spins. We have then:

$$\begin{array}{ccc} {\left||\psi |\right|}^{2} & = & \sum_{n=0}^{N}\;C\left(N,n\right)\;{\left|\hat{\psi }\left(n\right)\right|}^{2}=1,\\ E(\psi ) & = & \sum_{n}\sum_{\left\{\#S=n\right\}}{\left|\psi \left(S\right)\right|}^{2}\;E_{S}=\sum_{n}C\left(N,n\right){\left|\hat{\psi }\left(n\right)\right|}^{2}E_{n},\;\mathrm{where}\;E_{n}:=\sum_{\left\{\#S=n\right\}}E_{S}/C\left(N,n\right). \end{array}$$

Here “*S*” stands for a spin configuration, “#S” for how many “down” spins it contains and C(N,n) is the number of combinations with *n* spins down. Next we let

(26) $$\phi \left(n\right):=\sqrt{C(N,n)}\;\hat{\psi }\left(n\right),$$

and apply the theorem from above section, yielding:

(27) $$\int_{\left\{\;\sum \;|\hat{\psi }\left(n\right){|}^{2}=1\;\right\}}\;\exp\left\{-\;\sum \;C\left(N,n\right)\;|\hat{\psi }{\left(n\right)|}^{2}\;E_{n}\;\right\}=\frac{A_{\mathrm{sphere}}}{A_{\mathrm{ellipsoid}}}\;\int_{\left\{\;\sum \;|\phi \left(n\right){|}^{2}=1\;\right\}}\;\exp\left\{-{\;\sum \;|\phi \left(n\right)|}^{2}\;E_{n}\;\right\}.$$

Note that the prefactor in (27) is not in the integrand, not a function of ϕ, just a constant depending on *N*. Thus it plays no role in computing, e.g., the magnetization. With these preparatory remarks we can define our symmetric-wavefunction SCW (Schrödingerist Curie–Weiss) model. The magnetic energy associated with ϕ becomes:

(28) ECW(ϕ)..=..−1N∑n=0N|ϕn|2N−2n2.

Our SCW model is then defined by:

$$\begin{array}{c} {\left[\;F\left(\phi \right)\;\right]}_{\beta }=\int_{\left\{||\phi |{|}^{2}=1\right\}}d\phi \;\exp\left\{-\beta \;E_{CW}\left(\phi \right)\right\}\;\frac{F(\phi )}{Z},\;\;\;Z=\int_{\left\{||\phi |{|}^{2}=1\right\}}\;d\phi \;\exp\left\{-\beta \;E_{CW}\left(\phi \right)\right\}. \end{array}$$

The choice of the uniform base ensemble might be questioned; we leave this issue to Section 5.

### 4.1. The SCW Model with Wavefunction Energy

We observe that

(29) ECW(ϕ)..=..−Nm2(ϕ)+D(ϕ),

where

m(ϕ)=1N∑|ϕn|2N−2n;D(ϕ)=1N2∑|ϕ|2N−2n2..−..∑|ϕn|2N−2n2.

In passing, we point out the curious fact, revealed by (29), that D(ϕ) plays a role in a wavefunction model with the standard energy. We define:

(30) f..=..Nβ1−m2(ϕ)−D(ϕ)+NwD(ϕ).

Here we have added a term (Nβ) to make f≥0 and incorporated wavefunction energy. The intuition for the latter choice comes from the observation that, lacking that term, *f* can be small if *either* $m^{2}$ is large or *D* is large; incorporating the dispersion term with large enough wN, the last possibility should be suppressed.

As always, the model is defined by:

$$\begin{array}{c} {\left[\;m^{2}\;\right]}_{\beta }=\int_{{\left||\phi |\right|}^{2}=1}\;d\phi \;\exp\left\{-f\left(\phi \right)\right\}\;m^{2}\left(\phi \right)/Z;\;\;\;\;Z=\int_{{\left||\phi |\right|}^{2}=1}\;d\phi \;\exp\left\{-f\left(\phi \right)\right\}. \end{array}$$

Intuition suggests we should investigate cases were *w* is at least 1/N; hence, we define

(31) ω..=..Nw;

and we assume below that ω is a constant. This does not indicate a belief that *w* actually scales with *N*; if a wavefunction energy exists, then *w* is a constant of nature and does not scale. The role of the assumption is to avoid the suppression of all superpositions, as it would follow with fixed “*w*” in the mathematical limit of large *N* because of the factor $N^{2}$. This limit does not exist in reality; therefore our assumption is just a mathematical stratagem to prove theorems.

In Math Appendix A.4 we prove:

**Theorem 2.**  *Let positive numbers ω and ϵ satisfy:*

(32) $$\begin{array}{ccc} & & 1<\omega <4/3; \end{array}$$

(33) $$\begin{array}{ccc} & & \epsilon <\frac{1}{4}\;{\left(\;1+\sqrt{1-4\;r}\;\right)}^{2}; \end{array}$$

(34) r..=..ω−1ω.

Then there is a positive number $\beta_{c}=\frac{p^{*}\left(\epsilon \right)}{(\omega -1)\epsilon }$ such that, for $\beta >\beta_{c}$,

(35) $$\lim_{N\to \infty }\;{\left[m^{2}\right]}_{\beta }>\epsilon .$$

The factor $p^{*}\left(\epsilon \right)$ is a large deviation rate functional related to the application of the Gärtner–Ellis theorem; we do not write it here because it involves a technical discussion on large deviations theory, so we prefer to postpone it to Math Appendix A.4. The bound 4/3 is required for the application of the Gärtner–Ellis theorem but we expect that it has no meaning. With the specified range for ω, (33) always holds if ε<1/4.

The fact that at high temperature the magnetization is zero is in Appendix A.2, where we used a system of coordinates called polyspherical coordinates for a computation, see [30].

### 4.2. The SCW Model without Wavefunction Energy

Without WFE, we state this theorem:

**Theorem 3.**  *For the SCW model at any finite temperature (except T = 0) and for any ϵ>0:*

(36) $$\lim_{N\to \infty }{\;[\;\{\;|m\left(\phi \right)|>\epsilon \;\}\;]}_{\beta }=0.$$

Thus, the SCW model with the standard energy expression has no finite-temperature phase transition. Using large deviation techniques from probability theory, we reduced the proof of Theorem Three to a study of one real function of two variables, for which we supply exact formulas (see Math Appendix A.5). A property for the rate functionals, needed for the proof, is sustained by mathematical arguments and can be displayed, with some computational limits, via computer using statistical sampling from the domain of this function.

## 5. Ensemble Choice and Discussion

In previous sections, we constructed Gibbs ensembles where each state is weighted with a uniform measure on the sphere. A priori, we could consider other measures conserved by the Hamiltonian flows, e.g. modifications of the uniform one as dϕF(ϕ) where F(ϕ) satisfies {F(ϕ),E(ϕ)}=0 and the parenthesis {·} indicates the Poisson bracket. In the quadratic case this will be equivalent to [F,H]=0, where now [·] denotes the commutator. (This reflects the fact that there are many conserved quantities other than the energy and the norm in linear QM (including the moduli of the wavefunction component in the eigenstate directions), showing that linear QM is far from being ergodic.) As discussed in [19], Schrödinger’s equation and the non-linear modifications proposed there are simply Hamilton’s systems disguised; hence, Liouville’s theorem applies, implying that the base measure should be $\prod_{S}d\psi \left(S\right)$. The non-linear modification of WFE of course will eliminate the conserved quantities along eigenstate directions. Proving the ergodicity for some such modification would qualify the uniform measure as the unique choice for a base measure. But at the present time we can only demonstrate the existence of expanding and contracting directions in certain special cases, see [21].

An alternative ensemble, with the same base measure, could be constructed by adopting a Boltzmann factor that weights a state ψ taking into account the notion of possible microscopic states compatible with a macroscopic one. Concerning quantum theory, this notion can be tracked back to Einstein [31], in a paper about how to define quantum entropy. We think that, if the introduction of this modification has a meaning, it should be related to having considered indistinguishable particles jointly with a lack of spatial coordinates, which would add a degeneration on energy levels. For example, confining each spin in a spatial potential, we would have many levels for each spin and correspondingly many ways to arrange *n* spins down. In the SCW case, the proposal would be to replace in $\prod_{n}d\phi \left(n\right)$ with $\prod_{n}{\left[C\left(N,n\right)\right]}^{|\phi_{n}{|}^{2}}d\phi_{n}$, where C(N,n) is the number of states with *n* spins down. In this way, a wavefunction acquires a large combinatorical weight if amplitudes are concentrated on highly degenerated components.

Calling H the Hilbert space of wavefunctions of norm one decomposed as $H=\underset{n}{⊕}H_{n}$, where $H_{n}$ is the subspace of the wavefunctions with quantum number *n* and dimension $\dim H_{n}:=C\left(N,n\right)$, the proposed measure can be written also as

(37) $$\prod_{n}{\left[\dim H_{n}\right]}^{|\phi_{n}{|}^{2}}d\phi_{n}=e^{\left[\log\dim H](\phi \right)}\prod_{n}d\phi_{n},$$

with $\left[\log\dim H\right]\left(\phi \right)=\sum_{n}{\left|\phi_{n}\right|}^{2}\log\left(\dim H_{n}\right)$ and it is verified $\{\left[\log\dim H\right]\left(\phi \right),H_{SCWM}\left(\phi \right)\}=0$. Whether such a modified measure is still conserved by the Hamiltonian flow, with or without WFE, will depend on the precise details of the dynamics.

An a priori weight associated with a single wavefunction is questionable. However, at the moment we cannot exclude a different measure from the uniform one on the state space. Let us reflect on how this ensemble would introduce an analogous notion to the classical microcanonical entropy of the usual spin models. Introducing the energy and entropy densities, respectively,

$$e\left(\phi \right)=\langle \phi |e|\phi \rangle ,\;\mathrm{where}\;e\left(n\right)=-m_{N}^{2}\left(n\right)=-{\left(1-\frac{2n}{N}\right)}^{2},$$

$$s\left(\phi \right)=\frac{1}{\beta }\langle \phi |s|\phi \rangle ,\;\mathrm{where}\;s\left(n\right)=\frac{1}{N}\log C\left(N,n\right).$$

By Sterling, for large *N*, s(n) is approximated by

$$s\left(m_{N}\left(n\right)\right)=-\frac{1-m_{N}\left(n\right)}{2}\log\frac{1-m_{N}\left(n\right)}{2}-\frac{1+m_{N}\left(n\right)}{2}\log\frac{1+m_{N}\left(n\right)}{2}.$$

We also introduce

$$f^{\beta }\left(\phi \right):=e\left(\phi \right)-s\left(\phi \right).$$

The partition function becomes

(38) $$\begin{array}{c} \frac{1}{S_{N}}\int_{∥\phi ∥=1}e^{\log\dim H_{n}\left(\phi \right)}d\phi \;\;\exp\left(-\beta E\left(\phi \right)\right)\approx \frac{1}{S_{N}}\int_{∥\phi ∥=1}d\phi \;\;e^{-\beta Nf^{\beta }\left(\phi \right)}. \end{array}$$

Since we are interested in making *N* large, we used the approximation $\;\frac{1}{\beta N}\log\dim H_{n}\left(\phi \right)\approx s\left(\phi \right)$ and, neglecting the errors, we arrive at

(39) $$\begin{array}{c} Z_{N}=\frac{1}{S_{N}}\int_{∥\phi ∥=1}d\phi \;\;e^{-\beta N[-{\left(m\left(\phi \right)\right)}^{2}-s\left(\phi \right)-D\left(\phi \right)]}. \end{array}$$

The extra factor $e^{\beta Ns(\phi )}$ can be thought of as trying to restore a classical picture of a competition between internal energy and entropy due to high degeneracy of disordered macrostates. Anyway, since the role of this factor is giving large weights to some states of zero magnetization the WFE will be still necessary to define magnetic models.

The way to recover the usual ensembles may be to introduce $wN^{2}\;D$ with *w* constant (i.e., without keeping ω=wN constant) in the thermodynamic limit. In this case, one expects that the measure will concentrate on the set {ϕ:D(ϕ)=0}. Conceivably, we might arrive at the classical ensembles even in models without exchange symmetry, as considered in Section 3. However, apart from conflicting with a fundamental fact of quantum mechanics, i.e., that particles are indistinguishable and so wavefunctions must have symmetries, the ensemble will not magnetize in the thermodynamic limit even with suppression of superpositions, as already discussed after (22). We mention this is the route so far followed by various authors [32,33,34,35,36], where they reduced the ensembles on the sphere to the usual ones by introducing delta measures on eigenstates by different reasoning. The concentration we expect to be true for indistinguishable particles.

An interesting computation could be repeating the analysis of Theorem 3 with the modified measure (37), keeping first ω constant and in a second step considering the critical temperature with ω=wM in the limit of large *M*.

We guess that the uniform measure is still the right choice. The introduction of the Boltzmann factor appears a bit artificial, but it seems relevant when including $wN^{2}\;D$ with *w* constant, as otherwise the internal energy would have no competition, assuming the measure concentrates on eigenstates. However, we suggest there could be other meaningful pictures.

## 6. Conclusions

Once indistinguishable particles are considered, it seems we are forced to use a mean field type of interaction (plus the non-quadritic term of WFE to observe magnetization) because of the symmetry conditions on the wavefunctions at the moment we do not see a way to implement interactions such as nearest neighbors. (Current literature implementing nearest neighbor interactions on quantum states does not help, since they seem to apply the Hamiltonians without considering symmetry conditions.) To enrich our models we could consider to study the mean field for the Heisenberg model or considering the present model with a transverse magnetic field, which might be of interest as a model to study what are called quantum phase transitions [37]. Developing these ensembles could be of help to deal with problems for continuous models related to diffusion.

We might interpret the necessity of considering wavefunctions with symmetry conditions if we hope to have a thermodynamics that includes phase transitions as another justification of the usual quantum rules for combining indistinguishable systems.

Assuming the validity of Theorem Three (which claims that SCW with the usual energy does not spontaneously magnetize), our results suggest that blocking magnetic cats is related to phase transitions, presumably because states which are broad superpositions tend to have low magnetizations.

Perhaps the two main tasks for future investigations (which appear already very hard) are: improving the ability to compute spherical integrals, in the hope of deriving the functional dependence of magnetization on temperature; and generalizing the model to the case {−1,0,1} for symmetric wavefunctions and to the case {−1,1} for antisymmetric wavefunctions. We observe that these cases will automatically add degeneration to energy levels.

Also it might be worth considering *N*-levels for each spin, namely a pictorial way for replacing the discrete lattice with a confining potential for each spin and studying the limit for $wN^{2}$; this might help an understanding of Boltzmann entropy from a quantum perspective.

## Figures and Tables

**Figure A1:**
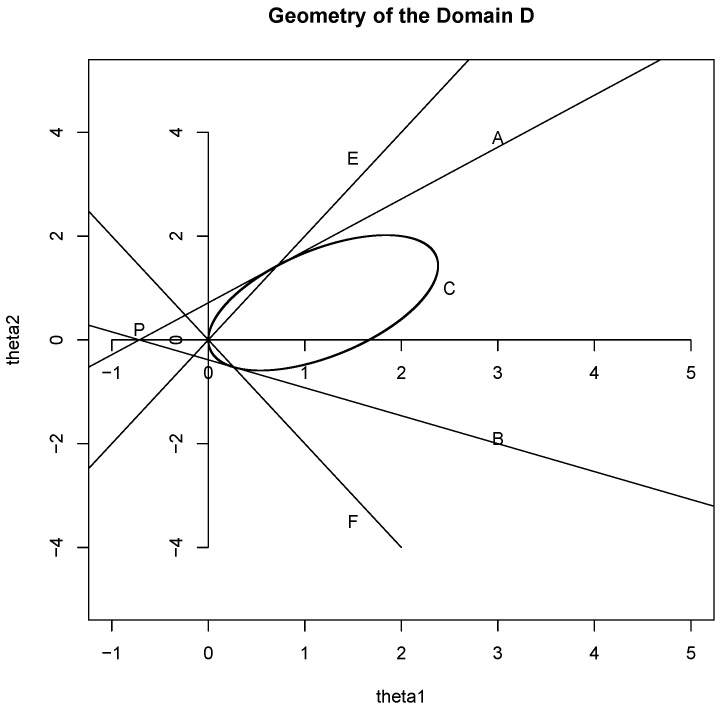
Regions used in describing the domain D where $c(\theta_{1},\theta_{2})$ is finite. These lines are obtained by the Tests above. Lines A and B are respectively $\theta_{2}=\theta_{1}\frac{x}{1-\varepsilon }+\frac{1}{2(1-\varepsilon )}$ from h(1)=1/2 and $\theta_{2}=-\theta_{1}\frac{x}{1+\varepsilon }-\frac{1}{2(1+\varepsilon )}$ from h(−1)=1/2. To the left of *P*, Test 1 and 2 can not be satisfied simultaneously. Lines E and F are respectively $\theta_{2}=2\theta_{1}$ from $y_{c}=1$ and $\theta_{2}=-2\theta_{1}$ from $y_{c}=-1$. The ellipse *C* is $4\theta_{1}^{2}\left(1-x\right)+\theta_{2}^{2}-4\theta_{1}\theta_{2}\varepsilon -2\theta_{1}=0$, obtained from $h(y_{c})=1/2$ of Test 3 to be applied for $\theta_{1}\geq 0$ (for $\theta_{1}<0$ Test 3 is satisfied).

**Figure A2:**
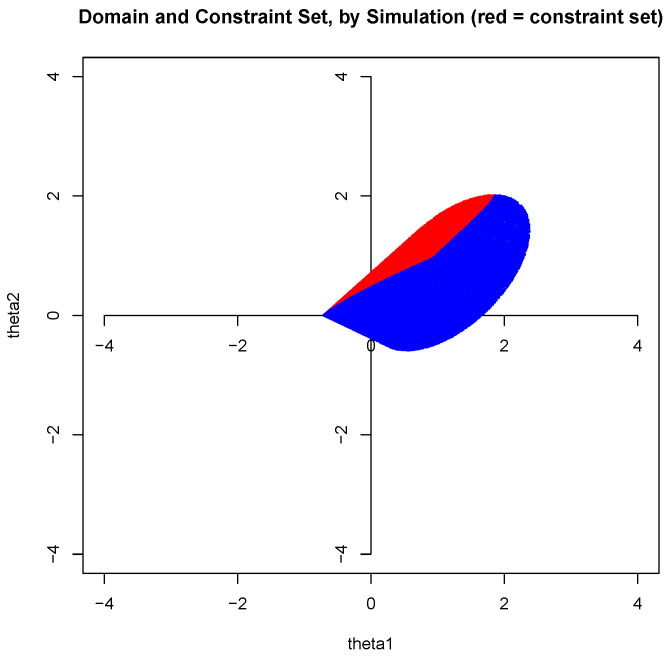
The domain *D*, in blue, defined in (A62) and the constraint set *G*, in red, defined in (A67) located by sampling.

**Figure A3:**
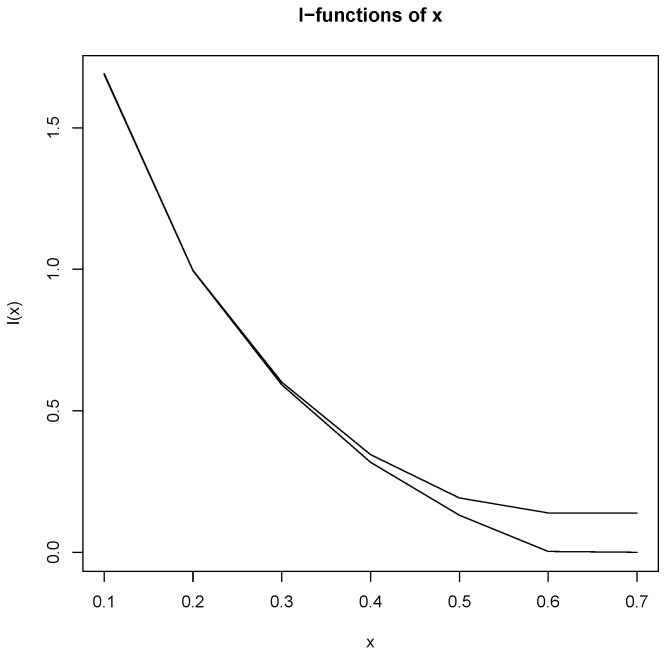
I-functions obtained by sampling. The upper one is the I-function appearing in the numerator.

## Data Availability

The work did not need data.

## Abbreviations

The following abbreviations are used in this manuscript:

COM	Center of mass
SCW	Schrödingerist Curie–Weiss
UIF	Useful integral formula
WFE	Wavefunction energy

## Appendix A

Here we collect in several appendices the various computational lemmas we used in the proofs that follow and the proofs themselves.

### Appendix A.1. Some Useful Lemmas

Let *S* be a compact manifold without boundary, f a real-valued function on *S*, dx a finite measure on *S*. Without loss of generality, we can take

(A1) ∫Sdx..=..|S|..=..1,

and assume f≥0. Let, for any bounded *g* on *S*:

$$\begin{array}{c} \left[g\right]=\int_{S}\;dx\;\exp\left\{-f\left(x\right)\;\right\}\;g\left(x\right)/Z,\;\;\;\mathrm{where}\;Z=\int_{S}\;dx\;\exp\left\{-f\left(x\right)\;\right\}. \end{array}$$

**Lemma A1** (Concentration Lemma)**.** *Let there be two open subsets of S, called U and V, and three positive numbers α, η and μ such that*

(A2) (A)……V⊂U(B)……f(x)≤η,…for…x∈V;(C)……f(x)≥α,…for…x∉U,(D)……|V|≥μ.

*Then:*

(A3)
[g]..=..R+ξ1+ζ,

*where*

(A4)
$$\begin{array}{ccc} R & =\int_{U}\;dx\;\exp\left\{-f\left(x\right)\;\right\}\;g\left(x\right)/Z_{U}, & Z_{U}=\int_{U}\;dx\;\exp\left\{-f\left(x\right)\;\right\};\\ \left|\xi \right| & \leq e^{-\alpha }\;e^{\eta }\;\mu^{-1}\;||g||, & \left|\zeta \right|\leq e^{-\alpha }\;e^{\eta }\;\mu^{-1}. \end{array}$$

Here ||g|| denotes the supremum norm of *g* on *S*. Note that R∈span{g(x):x∈U}. The intuition behind this lemma is the following. Suppose we are expecting the measure with density $\rho =e^{-f}/Z$ to be close to a delta function. Then this density must have a spike at the minimum of *f*, call it $\rho_{*}$, and the ratio of $\rho_{*}$ to values of ρ far away from the spike should tend to infinity. But we still will not get a delta function if the spike occupies a very tiny part of the manifold (imagine it as an infinitely thin needle) because it will contribute little to the integral. Hence the roles of the sets *U* and *V* and the bounds on *f* which prevent this “needle-like” behavior. The idea is that *V* is a small neighborhood of the global minimum of *f*. The minimum may not occur at a single point, but on a subset. The volume |V| should not be so small as to put a large factor in ξ and ζ, while α>η. Then ξ and ζ should tend to zero and the measure concentrates on the set *U*.

We note that the “balance of energy and entropy” game is contained in the difference α−η and in μ, measuring how *f* increases compared with the volume of the manifold required.

Next consider an integral of form:

(A5) $$\int_{B}\;\exp\left\{\;-f\left(\phi \right)\;\right\}\;d\phi ,$$

where dϕ stands for a probability distribution on a compact manifold with continuous density, *B* is a regular subset (non-empty interior and boundary of dϕ-measure zero) and *f* is a continuous function on *B* with values in [0,c] and level sets of zero dϕ-measure. Then we have the representation:

**Lemma A2** (Useful Integral Formula (UIF))**.**

(A6) ∫Bexp{−f(ϕ)}dϕ=e−c|B|..+..c∫01dxe−cxF(x),

*where*

(A7) F(x)..=..|f(ϕ)≤cx;…ϕ∈B|.

Here, |·| denotes the volume (“area”) of the subset *B*.

**Proof.** 

(A8) $$\begin{array}{ccc} & & \int_{B}\;\exp\left\{\;-f\left(\phi \right)\;\right\}\;d\phi \geq \sum_{k=0}^{K}\;e^{-kc/K}\;\left|\;\left\{\;\frac{(k-1)c}{K}\;<f\left(\phi \right)\leq \frac{kc}{K};\;\;\phi \in B\;\right\}\right|\;\\ & & \sum_{k=0}^{K}\;e^{-kc/K}\;\left\{\;F\left(\frac{k}{K}\right)-F\left(\frac{k-1}{K}\right)\;\right\}\approx \frac{1}{K}\;\sum_{k=0}^{K}\;e^{-kc/K}\;F^{\prime }\left(\;\frac{k}{K}\;\right) \end{array}$$

The last line tends, as K→∞, to:

(A9) $$\int_{0}^{1}\;dxe^{-cx}\;F^{\prime }\left(x\right).$$

We can get an identical upper bound in the limit K→∞ by substituting

(A10) e−(k−1)c/K..=..e−kc/Kec/K

in the calculation leading to (A8). Now the lemma follows from integration by parts, using F(0)=0 and F(1)=|B|. □

### Appendix A.2. A Curious Computation: The Average Magnetization at T = ∞

In this section we ask: what is the magnetization per spin at T = *∞*, meaning β=0? It should certainly be zero, which we prove here; the calculation will also provide some tools useful further on.

We adopt symmetric wavefunctions, which we denote by $\phi_{n}$, for n=0,…,N. Here *n* denotes the number of “down” spins. To avoid factors of 1/2 below we take our spins to have values ±1. In the following the expression: ∫dϕ will be understood to be an integral over the normalized measure on the sphere, i.e.,

(A11) $$\int \;d\;\phi =\frac{1}{A_{N}}\;\int_{||\phi ||=1}\;\prod \;d\phi_{n}.$$

where $A_{N}$ is the “area” of the 2N-sphere (the hypersphere in $R^{2N}$). We can write:

m2(ϕ)∞..=..M(ϕ)N2∞=∫dϕ∑n=0N|ϕn|2gn2;

where gn..=..1−2n/N. Developing:

(A12) m2(ϕ)∞..=..∑n=0N∑m=0N∫dϕ|ϕn|2|ϕm|2gngm=c1∑n=0Ngn2+c2∑n≠mNgngm,

where $c_{1}=\frac{1}{A_{N}}\;\int \;d\;\phi \;|\phi_{1}{|}^{4},\;\;\;\;\;c_{2}=\frac{1}{A_{N}}\;\int \;d\;\phi \;|\phi_{1}{|}^{2}\;{\left|\phi_{2}\right|}^{2}$. By adding and subtracting a term in (A12) and using $\sum_{n=0}^{N}\;g_{n}=0$, we can write:

(A13) m2(ϕ)∞..=..(c1−c2)∑n=0Ngn2.

The sum can be evaluated from formulas:

∑n=0Ngn2..=..N+1−4N∑n=0Nn+4N2∑n=0Nn2..=..N+1−4NN(N+1)/2+4N2N(N+1)(2N+1)6=13N+smallerorder.

We conclude that

(A14) limN→∞1N∑n=0Ngn2..=..1/3.

Putting in factors of *N* and 1/N in (A13) we deduce that we must consider the limit:

(A15) $$\lim_{N\to \infty }N\;\left(c_{1}-c_{2}\right).$$

By the same tricks but replacing $g_{n}$ by one, we find: 1=(N+1)c1..+..c2N(N−1), from which we conclude that $c_{1}$ = O(1/N) and $c_{2}$ = O($1/N^{2}$), so the issue becomes evaluating: $\lim_{N\to \infty }N\;c_{1}.$ Informally, this limit should be zero, since $|\phi_{n}{|}^{2}\approx 1/\left(N+1\right)$ on average, so $c_{1}$ should also be O($1/N^{2}$). To prove this rigorously, we resort to the polyspherical coordinate system: let ξ be a point on the (n − 1)-sphere (meaning the sphere in $R^{n}$). Write, see [30] Section 9.19,

(A16) ξ..=..ηsin(θ)..+..ζcos(θ),

where η lies on the (s − 1)-sphere and ζ lies on the (n − s − 1)-sphere. Then:

(A17) dξ..=..bnsins−1(θ)cosn−s−1(θ)dηdζdθ,wherebn..=..2Γ(n/2)Γ(s/2)Γ([n−s]/2).

We take s=2 and η as the first component of ξ and can therefore write:

(A18) ∫dξ|ξ1|4..=..bn∫0π/2dθsin(θ)cosn−3(θ)∫dηsin4(θ)|η|4∫dζ..=..bn∫dη|η1|4∫0π/2dθsin5(θ)cosn−3(θ).

Making the substitution u=cos(θ) the above integrand is a polynomial and the integral works out to be, substituting n=2N, 8n(n2−4)..=..1N(N2−1), also, the prefactor comes out:

(A19) b2N..=..2(N−1),

which proves the assertion about the order of $c_{1}$ and that the average magnetization at infinite temperature is zero in the thermodynamic limit.

This theorem can also be proved using LD theory but we omit it as it is covered in other Appendices.

### Appendix A.3. Models without Exchange Symmetries

For the computations of this section we add to the energy an external field with a constant λ, incorporate the factor of β, and take the positive version of the interaction energy (which merely multiplies *Z* by a factor). For example $\sum_{i,j;\;n.n.}^{N}\;{\left(\;S_{i}-S_{j}\;\right)}^{2}$ and $N+E_{WC}\left(\phi \right)$, respectively, for nearest neighbors and mean field. We write:

$$\begin{array}{c} E\left(\psi ;\lambda \right)=\beta \;\sum_{S}\;{\left|\psi \right|}^{2}\left(S\right)\;E\left(S\right)+\lambda \;M\left(\psi \right),\;\;\;M=\sum_{S}\;{\left|\psi \right|}^{2}\left(S\right)\;\sum_{i}\;S_{i}. \end{array}$$

Here and below “$\sum_{S}$”, resp. “$\prod_{S}$”, is shorthand for the sum, resp. product, over all configurations $\left\{\;S_{1}=\pm 1/2,\ldots ,S_{N}=\pm 1/2\;\right\}$. We will also write

(A20) E(ψ;λ)..=..∑S|ψ|2(S)ES(λ).

The starting point is to replace the spherical integral by a Gaussian integral, preserving the definition of the model by projecting ψ onto the sphere. Hence we can write:

$$\begin{array}{c} Z_{N}=c_{N}\;\int \;\prod_{S}\;d\psi_{S}\;\exp\left\{\;-{\left||\psi |\right|}^{2}/2-E\left(\psi /||\psi ||;\lambda \right)/2\;\right\}{,\;\;\;\;||\psi ||}^{2}=\sum_{S}\;{\left|\psi \right|}^{2}\left(S\right). \end{array}$$

We have introduced a factor of 1/2 before the energy to simplify some calculations below. The basic idea is to note that, although the integrand is singular at ψ=0, because the components of ψ are i.i.d. N(0,1), that value is improbable; indeed,

(A21) $$\sum_{S}\;{\left|\psi \right|}^{2}\left(S\right)\approx a_{N}=2\;2^{N}.$$

Therefore we define a simpler model by replacing the worrisome sum in the integrand by a constant:

(A22) Z^N..=..cN∫∏SdψSexp−||ψ||2/2−E(ψ;λ)/2aN;

which we will dub the “exactly solvable model” (ESM), as we are reduced to computing a Gaussian integral. We have:

(A23) Z^N..=..cN∏S∫dψSexp−||ψ||2/2σS2,whereσS2..=..1+ES/aN−1,

so from the basic Gaussian integral in two dimensions we find:

(A24) Z^N..=..∏S11+ES/aN.

Next we take two derivatives with respect to λ, denoted with primes, divide by ${\hat{Z}}_{N}$ and evaluate at λ=0:

(A25) $$\begin{array}{c} \frac{{\hat{Z}}_{N}^{\prime \prime }}{{\hat{Z}}_{N}}{|}_{\lambda =0}={\left\{\;\frac{1}{a_{N}}\;\sum_{S}\;\frac{M_{S}}{\left(1+E_{S}/a_{N}\right)}\;\right\}}^{2}+\frac{1}{a_{N}^{2}}\;\sum_{S}\;\frac{M_{S}^{2}}{{\left(1+E_{S}/a_{N}\right)}^{2}}. \end{array}$$

The first term is zero by the spin-flip symmetry ($M_{S}$ changes sign but since $E_{S}$ is quadratic in *S* it does not). Then, observing the prefactors of $1/a_{N}^{2}$, the whole thing tends to zero as N→∞ even without a factor of $1/N^{2}$, so there is no phase transition in the ESM. Finally we must estimate the difference in magnetizations in the two models. We can rewrite $Z_{N}$ as:

(A26) ZN..=..cN∫∏SdψSexp−||ψ||2/2−E(ψ)/2aN+χN,

where

(A27) $$\chi_{N}=\frac{E(\psi )}{{\left||\psi |\right|}^{2}}\;\left\{\;\frac{{\left||\psi |\right|}^{2}}{a_{N}}-1\;\right\}\;\mathrm{and}\;\xi_{N}=\frac{1}{4}\;{\left\{\;\frac{M(\psi )}{{\left||\psi |\right|}^{2}}\;\right\}}^{2}\;\left\{1-\;{\left(\;\frac{{\left||\psi |\right|}^{2}}{a_{N}}\;\right)}^{2}\;\right\}.$$

By expanding the exponential we can write

ZN≈Z^N..+..cN∫∏SdψSexp−||ψ||2/2−E(ψ)/2aNχN;ZN′′≈Z^N′′..+..cN∫∏SdψSexp−||ψ||2/2−E(ψ)/2aNξN..+..cN∫∏SdψSexp−||ψ||2/2−E(ψ)/2aNM(ψ)||ψ||22χN;

Hence, expanding to first order in the small quantities $\chi_{N}$ and $\xi_{N}$,

(A28) ZN″ZN≈Z^N″Z^N+cN∫∏SdψSexp−||ψ||2/2−E(ψ)/2aNξN/Z^N..−..cN∫∏SdψSexp−||ψ||2/2−E(ψ)/2aNM(ψ)||ψ||22χN/Z^N..−..Z^N″Z^N2cN∫∏SdψSexp−||ψ||2/2−E(ψ)/2aNχN.

Let us treat the third term first. Noting that

$$\begin{array}{c} {\left(\;\frac{M(\psi )}{{\left||\psi |\right|}^{2}}\;\right)}^{2}\leq \left(1/4\right)\;N^{2},\;\;\;\;\frac{E(\psi )}{{\left||\psi |\right|}^{2}}\leq \left(1/4\right)\;N. \end{array}$$

Applying Cauchy–Schwarz we can bound the term by:

(A29) $$\begin{array}{ccc} & & N^{3}/16\;{\left\{\;c_{N}\;\int \;\prod_{S}\;d\psi_{S}\;\exp\left\{\;-{\left||\psi |\right|}^{2}/2\right\}\;{\left\{\;\frac{{\left||\psi |\right|}^{2}}{a_{N}}-1\;\right\}}^{2}\right\}}^{1/2}\;\times \\ & & {\left\{\;c_{N}\;\int \;\prod_{S}\;d\psi_{S}\;\exp\left\{\;-{\left||\psi |\right|}^{2}/2-\;E\left(\psi \right)/a_{N}\;\right\}\;\right\}}^{1/2}/{\hat{Z}}_{N}. \end{array}$$

By standard CLT calculations, the first factor in curly brackets tends to zero, at rate $1/\sqrt{a_{N}}$. Using (A24) the second factor can be rewritten as ${\left\{\;\prod_{S}\;\frac{1+2E_{S}/a_{N}+E_{S}^{2}/a_{N}^{2}}{1+2E_{S}/a_{N}}\;\right\}}^{1/2},$ which is easily seen to be bounded (and in fact tends to one). In treating the second term we encounter instead the quantity ${\left\{\;c_{N}\;\int \;\prod_{S}\;d\psi_{S}\;\exp\left\{\;-{\left||\psi |\right|}^{2}/2\right\}\;{\left\{\;{\left(\;\frac{{\left||\psi |\right|}^{2}}{a_{N}}\;\right)}^{2}-1\;\right\}}^{2}\right\}}^{1/2}.$ This term can also be shown to tend to zero. E.g., writing “E” for the Gaussian integral, factoring the difference of squares and making another Cauchy–Schwarz, we have to estimate:

(A30) E∑|ψS|2aN−14..=..E1aN∑|ψS|2−14..=..1aN4∑S,S′,S″,S‴E|ψS|2−1···|ψS‴|2−1..=..6aN4∑SE|ψS|2−122..+..4aN4∑SE|ψS|2−14

which is O$\left(a_{N}^{-2}\right)$. We also have to estimate $E\;{\left\{\;\frac{{\left||\psi |\right|}^{2}}{a_{N}}+1\;\right\}}^{4}$, which by the same tricks is seen to be bounded. Recalling that

(A31) $$\frac{{\hat{Z}}_{N}^{\prime \prime }}{{\hat{Z}}_{N}}\;\to \;0,$$

the other term in (A28) is even easier to treat. Since moments of Gaussians increase more slowly than a factorial, the sum of the other terms is dominated by a convergent series and of smaller order. QED.

Now consider adding wavefunction energy:

(A32) EWFE(ψ)..=..wN2D(ψ);D(ψ)..=..1N2<ψ|∑Si−<ψ|∑Si|ψ>2|ψ>

(A33) ..=..1N2∑i,j<ψ|SiSj|ψ>−∑i<ψ|Si|ψ>2

Let us switch to the Gaussian version, divide ψ by ||ψ|| and replace ${\left||\psi |\right|}^{2}$ by $a_{N}$. Treating the second term in the last line above, define

(A34) X±,i..=..12aN∑S^|ψ|2(S1,…,±12,…,SN),

where “$\sum_{\hat{S}}$” means summation over the spin configurations with $S_{i}$ held at the fixed value indicated. Then we can write:

(A35) <ψ|Si|ψ>=1aN∑S|ψ(S)|2Si..=..X+,i−X−,i.

Note that, using E for expectation over the Gaussian distribution:

(A36) $$\begin{array}{c} \;E\left(\;X_{+,i}-X_{-,i}\right)=0,\;E{\left(\;X_{+,i}-X_{-,i}\right)}^{2}=E{\left(\;X_{+,i}-E\;X_{+,i}\right)}^{2}+E{\left(\;X_{-,i}-E\;X_{-,i}\right)}^{2}; \end{array}$$

and

(A37) EX+,i−EX+,i2..=..E12aN∑S^|ψ|2(S1,…,12,…,SN)−22.

Noting the average of i.i.d. mean-zero random variables this last is O$\left(1/a_{N}\right)$. The last term in (A32) is a sum of *N* terms which are not independent but all of the order just computed. By a simple Jensen inequality, the sum is at most *N* times this order. We conclude that the whole term is negligible. The first term is of the form of a mean-field model, still quadratic in ψ. Hence it can be added to the usual magnetic energy. The bound on $E_{S}$ increases to $N^{2}$, but does not affect the argument. For the comparison with the actual model with WFE, we encounter the expression

(A38) $$\frac{1}{4}\;\left\{\;\frac{M(\psi )}{{\left||\psi |\right|}^{2}}\;\right\}\;\left\{\;\left(\;\frac{{\left||\psi |\right|}^{2}}{a_{N}}\;\right)-1\;\right\},$$

which can be treated as before.

Thus the argument of the quadratic case goes through and yields that with WFE there is no magnetization at finite temperature.

### Appendix A.4. SCW with Wavefunction Energy

We prove here that, assuming the hypotheses of Theorem Two, if

(A39) $$\beta >\frac{{\underline{p}}^{*}\left(\epsilon \right)}{(\omega -1)\epsilon },$$

where ${\underline{p}}^{*}\left(\epsilon \right)$ is a certain positive function specified in the proof below, then the conclusion of the theorem follows. We will apply the Concentration Lemma (A3). We can assume by adding a term as usual to render *f* positive:

(A40) f..=..Nβ1−m2(ϕ)+(ω−1)D(ϕ).

We next define the quantities involved in the lemma. For the sets *U* and *V* we take, given two positive numbers ϵ and η (η may depend on *N*),

U..=..ϕ:…m2(ϕ)≥ϵ;V=f≤η.

Further, define:

α..=..βN1−ϵ.

We next check the hypotheses of the Concentration Lemma. A quick calculation gives the equivalent description of set *V*:

$$V=\left\{\;\phi :\omega \;m^{2}\left(\phi \right)\geq \left(\omega -1\right)\;\sum \;{\left|\phi_{n}\right|}^{2}\;g_{n}^{2}\;+\;\left(1-\eta /\beta N\right)\;\right\}=\left\{\;\phi :m^{2}\left(\phi \right)\geq \sum \;{\left|\phi_{n}\right|}^{2}\;a_{n}\;\right\},$$

where $g_{n}=1-\frac{2\;n}{N}$ and we have defined

an=ω−1ωgn2..+..1−η′/βω=rgn2..+..δ;

Since ω>1, assuming $\eta =\eta^{\prime }\;N$ and $1-\eta^{\prime }/\beta >\omega \;\epsilon$, the condition defining *V* implies V⊂U. Since ω>1, it follows from (A40) that f≥α on $U^{c}$. For the lower bound on |V|, we translate to a model with i.i.d. Gaussians, call them $\chi_{n}$, replacing:

(A41) $$\phi_{n}⟶\;\frac{\chi_{n}}{\sqrt{\sum |\chi_{n}{|}^{2}}},$$

and the definition of *V* becomes:

(A42) V..=..∑n=0N|χn|2gn2≥∑n=0Nan|χn|2∑n=0N|χn|2.

(Since $\psi_{n}$ has both a real and an imaginary part, we should double the lengths of these sums, but clearly this has no impact on the final result.) We have to find an exponential lower bound on P[V]. Since $a_{n}\geq \delta$, we can get a lower bound by writing:

PV≥P1N∑n=0N|χn|2gn2≥1N∑n=0Nan|χn|21N∑n=0Nanδ|χn|2..=..Pδ1N∑n=0N|χn|2gn≥1N∑n=0Nan|χn|2+Pδ1N∑n=0N|χn|2gn≤−1N∑n=0Nan|χn|2.

We will work on the first probability on the last line. (The second one is really the same since, multiplying the inequality through by −1, replacing $g_{n}$ by $-g_{n}$ just reverses the order of its values.) According to Gärtner–Ellis [38], we have to compute:

(A43) p(θ)..=..limN→∞1NlogEexp{θ∑χn2bn},

where E denotes expectation and

$$\begin{array}{c} b_{n}=\sqrt{\delta }\;g_{n}-a_{n}=\sqrt{\delta }\;\left(\;1-\frac{2n}{N}\;\right)-r\;{\left(\;1-\frac{2n}{N}\;\right)}^{2}-\delta , \end{array}$$

Note that $b_{n}$ can take negative and positive values; hence, θ must be restricted to ensure that the integral is finite. From the standard Gaussian integral we get:

(A44) p(θ)..=..−12limN→∞∑log1−2θbn,

which, provided the integrand is bounded, we recognize as the Reimann sum converging to:

p(θ)..=..−12∫01dulog1−2θA(1−2u),

where

A(x)..=..δx−rx2..−..δ.

By a change of variable this equals:

(A45) p(θ)..=..−14∫−11dxlog1−2θA(x).

The LD approach requires us to compute:

(A46) p*(y)..=..supθθy−p(θ);p_*=infy>0p*(y);

and then the asympotic lower bound is $\exp(-{\underline{p}}^{*}\;N\;)$ [38]. By the definition of the domain where p(θ)<+∞ in the Gärtner–Ellis theorem, the supremum over θ in the definition of $p^{*}\left(y\right)$ can be limited to:

(A47) $$\frac{1}{2\;A_{\min}}\leq \theta \leq \frac{1}{2\;A_{\max}},$$

where $A_{\min}$ is negative but we do not need to know it, while by a simple computation:

Amax..=..δ4−3ω4(ω−1).

(This is the max on the whole line.) We must have positive values of A(x) somewhere in the interval [−1, 1], for otherwise limp(θ)=−∞ as θ→∞, so $p^{*}\left(x\right)=+\infty$ for x≥0. This requires $A_{\max}>0$ and that the lower root of A(x)=0 lies in the interval [0, 1] (since A(0)=−δ<0 and $A^{\prime }\left(0\right)>0$). The root is easily computed to be:

x−..=..δ1−1−4r2r,

Since r<1/4, this root is real; letting $u=\sqrt{1-4\;r}$ the condition $x_{-}<1$ becomes

$$\delta <\frac{1}{4}\;{\left(\;1+u\;\right)}^{2},$$

Since we will eventually identify δ with ϵ, the above inequality is identical to (33). Since 0≤u≤1, the infimum of the right side is 1/4.

The problem of computing $p^{*}\left(x\right)$ is then well defined. We note that, if the point at which $A\left(x\right)=A_{\max}$ lies in the unit interval, then, as $\theta \to 1/2A_{\max}$, the integral looks as if it might diverge. However, it remains finite, because logarithmic singularities are integrable. e.g., ∫01dxlog(x)=limϵ→0∫ϵ1dxlog(x)=limϵ→0xlog(x)−xϵ1..=..−1, not −∞. However, the derivative $p^{\prime }\left(\theta \right)$ will go to infinity at the boundaries (Ellis calls such a function “steep” and it is an assumption of his theorem).

Assuming that $0<{\underline{p}}^{*}<\infty$, it will suffice for Theorem Two to know that for some $\eta^{\prime }>0$:

$$\begin{array}{ccc} {\underline{p}}^{*}+\eta^{\prime } & \leq & \beta \;\left(\;1-\epsilon \;\right);\;\eta^{\prime }\leq \beta \;\left(\;1-\omega \;\epsilon \;\right); \end{array}$$

We can define $\eta^{\prime }$ to saturate the second inequality above (note that ωϵ<1), which also yields δ=ϵ. The conclusion of the theorem follows.

Can we compute p(θ) and ${\underline{p}}^{*}\left(y\right)$? In fact, the integral defining p(θ) is elementary and can be computed as follows. First, integrate by parts:

p(θ)..=..−14∫−11dxlog1−2θA(x)=−14∫−11dxx′log1−2θA(x)..=..−14xlog1−2θA(x)−11−θ2∫−11dxxA′(x)1−2θA(x).

There are two cases, depending on whether the denominator in the integrand factorizes or not. For θ positive, it does not; hence, the denominator is an irreducible quadratic. For some negative θ it does factorize. We can proceed by elementary linear and trig substitutions, yielding either log(linear), log(quadratic), or arctan(quadratic) terms. Clearly, given such formulas with nonalgebraic functions, computing the supremum cannot be expected in closed form, so we would resort to the computer, but do not report detailed results here.

### Appendix A.5. SCW without Wavefunction Energy

The idea is to determine whether

(A48) $$\lim_{N\to \infty }\;\int_{m(\phi )\geq \epsilon }\;d\phi \;\exp\left\{\;-\;\beta \;E_{\mathrm{CW}}\;\right\}/Z=0,$$

or not, where

(A49) Z..=..∫dϕexp{−βECW}.

and similarily with {m(ϕ)≥ϵ} replaced by {m(ϕ)≤−ϵ}. We can rewrite the integrals as:

(A50) ∫m(ϕ)≥ϵdϕexp{−f}/Z,Z..=..∫dϕexp{−f},

where

$$\begin{array}{c} f=\beta \;N\;\left(\;1-\sum \;|\phi_{n}{|}^{2}\;g_{n}^{2}\;\right),\;\;\;\;g_{n}=1-2\;n/N. \end{array}$$

where as usual we have added a term so that 0≤f≤βN. To compare these integrals in numerator and denominator, we turn to the UIF, setting B={m(ϕ)≥ϵ} in the numerator and *B* equal to the whole sphere in the denominator. Thus, for the numerator we will have to estimate:

(A51) $$|\;\{\beta \;N\;\left(\;1-\sum \;|\phi_{n}{|}^{2}\;g_{n}^{2}\;\right)\leq \beta \;N\;x;\;\sum |\phi_{n}{|}^{2}\;g_{n}\geq \;\epsilon \;\}\;|,$$

which, replacing wavefunctions on the sphere by i.i.d. Gaussians, equivalently:

(A52) $$P\left[\;\left(1-x\right)\;\sum \chi_{n}^{2}\leq \sum \;\chi_{n}^{2}\;g_{n}^{2};\;\sum \;\chi_{n}^{2}\;g_{n}\geq \epsilon \;\sum \chi_{n}^{2}\;\right],$$

(since $\phi_{n}$ has two components, the sums are now over 2N + 1 rather than N + 1 indices, with the $g_{n}$ repeated; however, as we are taking the limit as N→∞ we do not bother with factors of two everywhere) which is the same as writing (introducing factors of 1/N for later purposes):

(A53) $$P\left[\;1/N\;\sum \chi_{n}^{2}\;\left(1-x-g_{n}^{2}\right)\leq 0;1/N\;\sum \;\chi_{n}^{2}\;\left(\;g_{n}-\epsilon \;\right)\geq 0\;\right].$$

To motivate the appeal to the Gärtner–Ellis theorem, let $P_{2;N}\left(x\right)$ stand for the probability of the set appearing above and $P_{1;N}\left(x\right)$ for the probability with the second restriction dropped. We are interested in the ratio:

(A54) $$p_{N}\left(x\right)=\frac{P_{2;N}\left(x\right)}{P_{1;N}\left(x\right)},$$

which has the interpretation of the conditional probability that the magnetization is greater than ϵ, given a bound “*x*” on the energy. We expect that

(A55) $$\lim_{N\to \infty }p_{N}\left(x\right)=0.$$

Then, integrating over *x* (as in the UIF) we shall arrive at the result. Noting that the events considered have the form of averages of random variables whose means lie outside the indicated bounds, we expect large-deviation asymptotics for both, and so it is natural to consider

(A56) $$\begin{array}{c} \lim_{N\to \infty }-\frac{1}{N}\log\;p_{N}\left(x\right)=\lim_{N\to \infty }-\frac{1}{N}\log\;P_{2,N}\left(x\right)+\lim_{N\to \infty }\frac{1}{N}\log\;P_{1;N}\left(x\right) \end{array}$$

and to treat the two terms separately and then compare. We used $\lim_{N}1/N\log P_{N}\left(S\right)=-I\left(S\right)$ with $I\left(S\right)=\inf_{S}I\left(y\right)$ for the involved sets *S*. But what a large deviation principle gives are upper and lower bounds. To have equality $\lim_{N}1/N\log P_{N}\left(S\right)=-I\left(S\right)$ we need *S* to be *I*-continuous, i.e., I(int(S))=I(cl(S)), see page 30 [39]. This is the case.

To implement the Gärtner–Ellis procedure, we introduce the random vector with two components:

(A57) YN..=..1/N∑χn2(1−x−gn2);1/N∑χn2(gn−ϵ),

so we are interested in

(A58) $$P\left[\;Y_{N}\in \left[\;\mathrm{Upper}\;\mathrm{left}\;\mathrm{quadrant}\;\mathrm{of}\;R^{2}\;\right]\;\;\right].$$

To apply G-E we must compute:

(A59) c(θ1,θ2)..=..limN→∞1NlogEexp{NYN;1θ1+YN;2θ2}..=..limN→∞−12N∑log{1−2θ1(1−x−gn2)+θ2(gn−ϵ)}..=..−12∫−11dylog{1−2θ1(1−x−y2)+θ2(y−ϵ)}..=..−12∫−11dylogq(y);

where

(A60) q(y)..=..1−2θ1(1−x−y2)+θ2(y−ϵ)..=..2θ1y2−2θ2y+b,

where *b* is a constant depending on the thetas.

We must first determine the region in the $\left(\theta_{1},\theta_{2}\right)$ plane in which $c(\theta_{1},\theta_{2})<+\infty$. Defining

(A61) h(y)..=..h(y;θ1,θ2)=θ1(1−x−y2)+θ2(y−ϵ);

this region, call it *D*, is defined by:

(A62) D..=..(θ1,θ2):h(y)≤12…forally,−1≤y≤1.

We observe that *D* is the domain where $c(\theta_{1},\theta_{2})$ is finite, related to the conditions for the validity of the Gärtner–Ellis theorem, not the domain of the logarithm argument. The geometry of this region is a bit complicated. These are the tests for whether a point lies in *D*:

Test1: h(1)≤1/2;

Test2: h(−1)≤1/2;

Test3: if $y_{c}=\theta_{2}/2\theta_{1}$, the critical point of h(y), lies in [−1, 1], then test whether $h(y_{c})\leq 1/2$.

Now see Figure A1. By tests one and two, *D* lies between the lines A and B, and to the left of point P. Test three applies in between lines E and F; to the right of the axis, it is satisfied within the oblique ellipse, curve C; to the left of the axis, *h* is negative. Hence we obtain a diamond-shaped compact region of the plane.

We note that $c(\theta_{1},\theta_{2})$ is convex (by examination of its Hessian matrix, omitted) and “steep”, meaning its derivatives approach infinity at the boundaries of *D*. We have an ellipse as in Figure A1 if $\left(1-x\right)-\epsilon^{2}>0$, otherwise a hyperbola. We restrict the study to the ellipse case, since it is the relevant one: we are primary interested in small ϵ>0 and, from the proof after (A105), one can see that the small *x* are the relevant ones for low temperatures. Therefore, given ϵ, we always restrict to the *x* small enough to satisfy $\left(1-x\right)-\epsilon^{2}>0$.

The G-E procedure is to compute the dual function:

(A63) f(z1,z2)..=..supθ1,θ2z1θ1+z2θ2−c(θ1,θ2);

from which you can compute the I-function as:

(A64) I2..=..I2(x;ϵ)..=..inff(z1,z2):z1≤0;z2≥0.

By the convexity and steepness of *c*, the supremum in (A63) is attained at a critical point for which

(A65) ∂c∂θ1..=..z1;∂c∂θ2..=..z2;

so looking forward to a possible computer search (for which we do not want to solve equations but rather evaluate formulas), we define

(A66) k(θ1,θ2)..=..∂c∂θ1θ1..+..∂c∂θ2θ2−c(θ1,θ2),

Let *G* denote the constraint set in the $\left(\theta_{1},\theta_{2}\right)$-plane:

(A67) G..=..(θ1,θ2):∂c∂θ1≤0;∂c∂θ2≥0.

We can express the I-function as

(A68) I2..=..I2(x;ϵ)..=..infk(θ1,θ2):(θ1,θ2)∈G∩D;.

For the proof we have to compare the I-function for the two-variable, two-inequality problem with that of the one-variable, one-inequality problem, given by

(A69) I1..=..I1(x)..=..infk(θ1,0):θ1∈L;∂c∂θ1(θ1,0)≤0,

where *L* is the part of the horizontal axis that lies in *D*. A first, critical question to ask is whether necessarily $I_{2}\leq I_{1}$ because the set over which we compute the infimum for $I_{2}$ contains the set for which we compute the infimum for $I_{1}$. However, this is not the case. We have:

(A70) ∂c∂θ2..=..∫−11dyyq(y)..−..ϵ∫−11dy1q(y),

where q(y)=1−2h(y)≥0 by the restriction that $(\theta_{1},\theta_{2})\in D$. The first integral can be done easily by elementary calculus:

(A71) ∫−11dyyq(y)..=..14θ1∫−11dy4θ1y−2θ2+2θ22θ1y2−2θ2y+b..=..14θ1log2θ1−2θ2+b2θ1+2θ2+b+θ22θ1∫−11dy1q(y),

Now note that, at $\theta_{2}=0$, this integral is zero, and so is the first term in (A70), while the second term there is negative. Hence $\left\{\;\partial c/\partial \theta_{2}\geq 0\;\right\}$ is disjoint from *L*.

We need a formula for the first partial of *c*:

(A72) ∂c∂θ1..=..∫−11dy1−x−y22θ1y2−2θ2y+b.

By long division we can write:

(A73) 1−x−y22θ1y2−2θ2y+b..=..A..+..B+Cyq(y),

where

(A74) A=−12θ1;…B=1−x−Ab;…C=2θ2A.

Hence:

(A75) ∂c∂θ1..=..∫−11dyA..+..B∫−11dy1q(y)..+..C∫−11dyyq(y)..=..2A..+..Cθ22θ1+B∫−11dy1q(y)..+..C4θ1logq(1)q(−1).

where we have plugged in (A71) for one integral.

Note that we have reduced computing these derivatives to computing one integral, of 1/q(y) over the interval [−1,1]. There are two cases, depending on whether q(y) is irreducible or factorizes. The discriminant is:

disc...=..4θ22−8θ1b..=..4θ22−8θ11−2θ1(1−x)−θ2ϵ.

If disc. >0, *q* factorizes as

(A76) $$q\left(y\right)=2\theta_{1}\;\left(y-r_{+}\right)\;\left(y-r_{-}\right),$$

where the two roots are outside the interval [−1,1]. The integrals can then be performed by partial fractions, yielding logarithmic terms:

(A77) ∫−11dy1q(y)..=..12θ1(r−−r+)log(1−r−)(1+r+)(1−r+)(1+r−).

If disc.<0, in our case *q* can be written:

(A78) $$q\left(y\right)=A^{\prime }{\left(y-B^{\prime }\right)}^{2}+C^{\prime },$$

with $A^{\prime }>0$ and $C^{\prime }>0$, and one makes a trig substitution:

(A79) tan(u)..=..A′C′(y−B′).

The result contains inverse trig functions:

(A80) ∫−11dy1q(y)..=..1A′C′arctanA′C′(1−B′)..−..arctanA′C′(−1−B′).

To check these calculus formulas (and our computer implementation of them) we computed the integral directly (numerically) using the trapezoid rule and compared. We also need a formula for *c*, which we can obtain by integrating by parts:

(A81) c..=..−12∫−11dylogq(y)..=..−12∫−11dyy′logq(y)c..=..−12logq(1)q(−1)..+..12∫−11dyyq′q

Here

(A82) 12∫−11dyyq′q..=..∫−11dy2θ1y2−θ2y2θ1y2−2θ2y+b,

and applying long division again:

(A83) 2θ1y2−θ2yq..=..A..+..By+Cq,

with

(A84) A=1;…B=θ2;C=−b.

Thus we get

(A85) 12∫−11dyyq′q..=..2..+..θ2∫−11dyyq..−..b∫−11dy1q.

Combining with previous results yields:

(A86) c..=..2−12logq(−1)q(1)+θ24θ1logq(1)q(−1)+θ222θ1−b∫−11dy1q.

For the function *k* we can compute from its definition, but also some simplifications accrue:

(A87) k(θ1,θ2)..=..∂c∂θ1θ1..+..∂c∂θ2θ2−c(θ1,θ2)..=..∫−11dyθ1(1−x−y2)q(y)..+..θ2(y−ϵ)q(y)..−..c(θ1,θ2)..=..∫−11dyh(y)1−2h(y)−c(θ1,θ2)..=..−12∫−11dy−2h(y)+1−11−2h(y)−c(θ1,θ2)..=..−1..+..12∫−11dy1q(y)..+..12∫−11dylog(q(y)).

For the theorem we have to evaluate the asymptotics of the ratio:

(A88) $$\frac{\exp\left\{\;-N\;\beta \;\right\}\;|B|+N\;\beta \;\int_{0}^{1}\;dx\exp\left\{\;-N\;g_{2}\left(x\right)\;\right\}}{\exp\left\{\;-N\;\beta \;\right\}+N\;\beta \;\int_{0}^{1}\;dx\exp\left\{\;-N\;g_{1}\left(x\right)\;\right\}},$$

where *B* is the set appearing in (A53) and

(A89) g1,2(x)..=..βx+I1,2(x).

We next list some properties of the *I*-functions that will imply the theorem’s conclusion:

Assumptions on $I_{2}\left(x\right)$ and $I_{1}\left(x\right)$

(A90) (a)…I2(x)>I1(x)…for…x∈(0,1];(b)…I1,2…aredifferentiable,monotonedecreasingandconvexon(0,1];(c)…limx→0I1,2(x)=+∞;(d)…I2(1)>0;(e)…I1(x)=0…for…x∈[2/3,1].

The rate functionals are defined as

(A91) I1,2(x)..=..−limN→∞1/NlogP1,2;N(x).

Since they are computed from independent Gaussian variables, even if not identically distributed [40], the corrections to the large deviations are $O\left(N^{-1/2}\right)$:

(A92) $$P_{1,2;N}\left(x\right)=\exp\left[-N\left(I_{1,2}\left(x\right)+O\left(\frac{\log N}{N}h_{1,2}\left(x\right)\right)\right)\right].$$

Therefore the approximation $P_{1,2;N}\left(x\right)\approx \exp\left[-NI_{1,2}\left(x\right)\right]$ is very good for large *N*. If $I_{2}\left(x\right)\neq I_{1}\left(x\right)$ then it has to be $I_{2}\left(x\right)>I_{1}\left(x\right)$. The study of their large deviation corrections should prove it.

For (a) we know from the inequality $P_{2;N}\left(x\right)<P_{1;N}\left(x\right)$ (for fixed *N*) and the Gärtner–Ellis Theorem $I_{2}\left(x\right)\geq I_{1}\left(x\right)$, and we showed that these functions result from infimums of the same function over different sets; therefore, it is implausible that they are exactly equal for all *x* smaller than a given *x**. (Because of the property (b) and $I_{2}\left(x\right)\geq I_{1}\left(x\right)$, if there exists a $x^{*}$ such that $I_{1}\left(x\right)=I_{2}\left(x\right)$, then they have to coincide for all *x* smaller than $x^{*}$. We supply some evidence that it is not so, from numerical approximations, see the Computational Appendix A.6 and Figure A3.) From the approximation $P_{1,2;N}\left(x\right)\approx \exp\left[-NI_{1,2(x)}\right]$ and $P_{i;N}\left(x^{\prime }\right)>P_{i;N}\left(x\right)$ for $x^{\prime }>x$ one expects strict decreasing monotonicity. This is indeed what we observed from the computer computations. Differentiability and strict decreasing monotonicity can be deduced from the Envelope Theorem in [41] at pag. 605 applied to (A63) and (A64). $f(z_{1},z_{2})$ is strictly convex and also the Lagrangian $f(z_{1},z_{2})-\lambda \cdot z$ to optimize if the optimizer is on the boundaries. Applying the Envelope Theorem, on the right hand side of (A93) the derivative coincides with that of *f* because the constraint functions do not have dependence on *x*. From $\underset{z}{\inf}\;\;f\left(z_{1},z_{2}\right)$, calling $\theta^{*}\left(z\right)$ the solution of (A63) and $z^{*}$ the one of (A64), we have

(A93) $$\begin{array}{c} \frac{dI_{i}}{dx}=\frac{\partial f(\theta^{*}\left(z^{*}\left(x\right),x\right),z^{*}\left(x\right),x)}{\partial x}. \end{array}$$

The smoothness of $\theta^{*}$ and $z^{*}$ comes from the implicit function theorem. The right hand side gives $\theta_{1}^{*}\int_{-1}^{1}\frac{dy}{q}$, since q>0 we have that it is negative if and only if $\theta_{1}<0$. We know that $I_{i}\left(x\right)$ is decreasing by definition so it can be only $\theta_{1}<0$ or $\theta_{1}=0$, for $I_{1}\left(x\right)$ this second case gives $I_{1}\left(x\right)=0$, which it can be only for x≥2/3 by definition; therefore, $\theta_{1}^{*}<0$ when $I_{1}\left(x\right)$ is not trivial. For $I_{2}\left(x\right)$ the value $\theta_{1}^{*}$ stays in disjoint set from the axis $\theta_{1}=0$; therefore, it can be only $\theta_{1}^{*}<0$ since $I_{2}\left(x\right)$ is decreasing by definition. Second order differentiability needs to develop second order Envelope Theorems; of course it will not be $\frac{d^{2}I_{i}}{dx^{2}}=\frac{\partial^{2}f}{\partial x^{2}}$. Differentiability of the rate functionals can also be deduced from differentiability of the probability functions $P_{i;N}\left(x\right)$; in our case one can apply corollary 4.1 of [42] and corollary 32 of [43] because of the quadratic structure of the $\chi_{n}$’s and other conditions that are verified. These results should be extended to second order differentiability without problem [44]. We cannot deduce analytically the convexity property (varying *x* we are restricting in a continuous way the integration on a regular set of a Gaussian integral) and we observe this property from the numerical computations of the exact formulas of the rate functionals, see Appendix A.6, within the limits of our computer analysis, that is below x=0.1. Here we cannot obtain good numerical estimates since the sampling region shrinks to an infinitesimal volume and we have a vertical asymptote, as proved later for (c). In this vertical asymptote part convexity is natural. Anyway, from the proof after (A105), if there is some flex from some unexpected reason, the computations can be applied for smaller *x* (i.e., lower temperature) where the functionals are convex. From (A53) we have (d) since the first event for x=1 is the sure event, while the second never contains the average for any ε>0 (see also the Computational Appendix A.6 for a numerical computation).

Furthermore, (e) follows from a computation in a previous section, see (A14), and the remark that LD results from probabilities of sets that do not contain the mean; otherwise, by the Central Limit Theorem such probabilities go to one.

That leaves (c). It would follow immediately from the observation that $P_{1,2}\left(0\right)=0$ (since $g_{n}\leq 1$) if we can assume that two limits can be interchanged. As that is not obvious, we supply a proof using previously obtained formulas. Since certainly $I_{2}\left(x\right)\geq I_{1}\left(x\right)$ it suffices to prove (c) for the latter. Therefore, we set $\theta_{2}=0$ in the following. From (A75) we note that

(A94) ∂c∂θ1..=..1θ1−1+12∫−111q(y).

Let us define for a given *x*:

(A95) H..=..H(θ1)..=..−1+12∫−11dy1q(y).

We note H(0)=0, since q=1 there, and

(A96) ∂H∂θ1(0)..=..−12∫−11dy2y2−2(1−x)..=..43−2x;

so, if x<2/3, $\partial H/\partial \theta_{1}>0$

Furthermore:

(A97) ∂2H∂θ12..=..∫−11dy2y2−2(1−x)2q(y)3≥0,

since by definition of the domain D, q(y)≥0 for all *y* in [−1, 1]. Hence, *H* is convex.

Now consider the left-most point of the domain D on the horizontal axis, labeled “P” in Figure A1, which has $\theta_{1}$ coordinate −1/2x. By definition, as $\theta_{1}\to P$, q(y)→0 for some y∈[−1,1] (here, at the endpoints y=±1), so H→∞. Thus $H(\theta_{1})$ must have a second zero on the line, to the right of *P*; let us label it *Q*.

The constraint set is H≥0, so from (A94) we conclude that it lies entirely to the left of *Q* (and of course to the right of *P*).

Some computer work indicates that *Q* is very close to *P* for small *x*, which explains why we could not locate the constraint set by sampling for x<0.1. Hence, suppose that Q(x)=P(x)+δ(x) with δ(x) bounded above (or even, as we shall see, tends to zero) as x→0. From (A87) we have

(A98) k(θ1,0)..=..H+12∫−11dylog{q(y)}.

Note that, substituting −1/2x+η for $\theta_{1}$,

(A99) q(y)..=..1−y2x+2ηy2−2η(1−x).

Therefore, in the constraint set with the above assumption η≤δ(x), so the second term in *k* goes to infinity as x→0. Hence, since the first term is nonnegative by the constraint,

(A100) $$\inf\;\{\;k\left(\theta_{1},0\right):\;\theta_{1}\;\in \;D\;\}\to \infty ,$$

as x→0, which yields (c).

In order to prove that Q(x)→P(x) we apply the exact formula for the integral appearing in $H(\theta_{1})$, which, with $\theta_{2}=0$, lies in the factorizable case, see (A77). Letting

(A101) $$s=\sqrt{\;\frac{1}{-2\theta_{1}}+1-x\;},$$

we find

(A102) H..=..s2+x−14slog(1+s)2(1−s)2−1.

Next, let t=s−1, so that t=0 corresponds to $\theta_{1}=-1/2x$; i.e., P(x)=Q(x). Rewriting the equation for the root H=0 gives:

(A103) log(t)..=..log(2+t)−2(t+1)t2+2y+x;

so exponentiating both sides:

(A104) $$t=\left(2+t\right)\;\exp\left\{\;\frac{-2(t+1)}{t^{2}+2t+x}\;\right\}.$$

Note that, if *x* is small, the right-hand side of (A104) is very small at t=0 and remains so up to small values of *t*, while the left-hand side follows *t*. For instance, if x=0.01, up to t=0.01 the RHS is no more than about exp{−66} which is infinitesimal while the LHS reaches 0.01. Thus, as x→0, the solution of (A104) goes to zero, and hence Q(x)→P(x) (in fact exponentially fast), which implies (c).

Granted these assumptions, we now proceed to the proof of Theorem Two. Property (e) implies that we can replace the denominator in (A88) as follows:

(A105) $$\frac{\exp\left\{\;-N\;\beta \;\right\}\;|B|+N\;\beta \;\int_{0}^{1}\;dx\exp\left\{\;-N\;g_{2}\left(x\right)\;\right\}}{\exp\left\{\;-\left(2/3\right)\;N\;\beta \;\right\}+N\;\beta \;\int_{0}^{2/3}\;dx\exp\left\{\;-N\;g_{1}\left(x\right)\;\right\}},$$

Next, consider whether the *g*-functions have critical points in the intervals (0, 1) or (0, 2/3). Using a hat to denote those points, we are asking whether solutions exist for:

(A106) β+I1′(x^1)=0;……β+I2′(x^2)=0,……

First, suppose both exist. If so, since *g* is convex, the Laplace approximation applies yielding:

(A107) $$\int \;\exp\left\{\;-N\;g\left(x\right)\;\right\}\approx \frac{\sqrt{2\;\pi }\;\exp\left\{-N\;\hat{g}\;\right\}}{\sqrt{N\;{\hat{g}}^{\prime \prime }}},$$

where a hat means evaluate at the critical point.

We are now prepared to argue that the limit of the ratio in (A105) is always zero. There are four cases, defined by whether the “winner” (largest term asymptotically) in numerator and denominator is the first or second term:

Case: numerator, second term; denominator, second term. As the $\hat{g}$’s are minimums, ${\hat{g}}_{2}>{\hat{g}}_{1}$ and the ratio tends to zero.
Case: numerator, first term; denominator, second term. Then ${\hat{g}}_{1}<2/3\;\beta$ and we are looking essentially at the limit of
  (A108) $$\frac{\exp\{-N\;\beta \;\}\;|B|}{\exp\{-N\;{\hat{g}}_{1}\;\}}$$
  which clearly goes to zero.
Case: numerator, second term; denominator, first term. Now we are looking at
  (A109) $$\frac{\exp\{-N\;{\hat{g}}_{2}\;\}\;}{\exp\{-(2/3)\;N\;\beta \;\}}$$
  which goes to zero if ${\hat{g}}_{2}>2/3\;\beta$. The reverse is impossible, as the ratio would tend to infinity while we know it is bounded by one from the original definition.
Case: numerator, first term; denominator, first term. The ratio tends to zero.

Since we are interested in low temperatures (that is small *x*) the presented cases are the relevant ones. However, we can still ask: do these critical points exist? With our assumptions we know that

(A110) $$\lim_{x\to 0}\;I_{1,2}^{\prime }\left(x\right)=-\infty .$$

If in addition we knew that

(A111) limx→1I2′(x)=0…and…limx→2/3I1′(x)=0

we can conclude the c.p.’s exist. If not, for sufficiently small β a c.p. might not exist. For instance, let us postulate that $I_{2}^{\prime }\left(1\right)=-c<0$ or $I_{1}^{\prime }\left(2/3\right)=-c<0$. Then in either case

(A112) $$g^{\prime }\left(x\right)\leq \beta -c<0$$

for all *x* in the relevant interval. In this case *g* is monotonic and we can make a change of variable: u=g(x) to obtain

(A113) βN∫01dxexp{−Ng(x)}..=..βN∫g(1)g(0)du1−g′∘g−1(u)exp{−Nu}≤βN∫g(1)g(0)du1c−βexp{−Nu}

Therefore, for the numerator the integral is:

(A114) $$\leq \left[\;\frac{1}{c-\beta }\;\right]\;\exp\left\{-N\;\left[\beta +I_{2}\left(1\right)\right]\;\right\}$$

(where we have used assumption (c) to drop a term) and so, if $I_{2}\left(1\right)>0$, the first term in the numerator wins. In the denominator, the order is at most exp{−2/3βN}, so the numerator tends to zero faster whatever term predominates in the denominator.

### Appendix A.6. Computational Appendix

After implementing the functions appearing in Math Appendix A.5 in a program, we employed a sampling scheme to locate the constraint set. The idea is to choose at random a ray emanating from point “P” of Figure A1, then a point on the ray staying within the domain *D*. (We also tried a ray emanating from the origin, which gave similar results.)

By experiment, we discovered that for *x* near zero the region G shrank to nearly a line close to line A in Figure A1, while the constraint set for generating $I_{1}$ lay almost at the left endpoint, “P”. Hence, we needed sampling schemes that could be biased to prefer points near the endpoints of a given interval [a,b] of the real axis. Such schemes are given by the following: to bias near b, choose a point “*s*” by the scheme

(A115) s..=..a+log(zηu+1)/ηz..=..(exp{η(b−a)}−1)/η.

and to bias near a:

(A116) s=a−log(1−zηuexp{−η(b−a)})/η,

with the same expression for *z*. Plugging in a uniform random variable (produced by the system RNG) for *u* yields the sampling scheme for s∈[a,b]. With η=0 the sampling is uniform on the interval; large positive η yields bias. We will write: s=biased-sample(a,b) for the random sample.

Our sampling scheme in the region *D* was the following:

(1) Choose α: α=biased-sample(−x/(1+ϵ),x/(1−ϵ));

(2) Choose $\theta_{1}$: $\theta_{1}=\mathrm{biased}-\mathrm{sample}\left(\;-1/\left(2x\right),5/\left(1-x\right)\;\right)$;

(3) Let $\theta_{2}=\alpha \;\left[\theta_{1}+1/\left(2x\right)\right]$.

If the selected $\left(\theta_{1},\theta_{2}\right)$ pass all the tests to lie in *D*, accept the values; otherwise, reject them. To locate the constraint set, we sampled many pairs $\left(\theta_{1},\theta_{2}\right)$ as above and evaluated the two partial derivatives of *c*, keeping and plotting the points that had the required signs. The results—see Figure A2; parameters in the figure were x=0.7 and ϵ=0.3—show that *G* lies in the upper half of the plane and is disjoint from the horizontal axis. Figure A3 shows curves of the I-functions obtained by sampling for a few values of *x* (0.1 to 0.7, in increments of 0.1). We used 10 billion samples at each *x*-value. Unfortunately, we were unable to estimate the I-functions for x<0.1 because few or no sampled points fell in the constraint set (even with various choices of bias), for reason indicated earlier.
